# Supplementary material for: Trichoderma asperellum L. Coupled the Effects of Biochar to Enhance the Growth and Physiology of Contrasting Maize Cultivars under Copper and Nickel Stresses
Source: Plants (Basel). 2023 Feb 20;12(4):958. doi: 10.3390/plants12040958 (PMC9960312; doi:10.3390/plants12040958)
Supplement: Supplementary file 1 [file plants-12-00958-s001.zip › plants-2175106-supplementary.pdf]

Table S1: pH, electrical conductivity (EC- (mScm[-1])) soil moisture% and WHC-Water holding capacity (ml/L) of soil of *Zea mays* L. plant were determined under different treatments against copper and nickel stress.

Variety	Spring Corn									Footer Corn											
	T1	T2	T3	T4	T5	T6	T7	T8	T9	T10	T11	T1	T2	T3	T4	T5	T6	T7	T8	T9	T10	T11
pH	7.47±0.2b	6.15±0.1i	6±0.01j	7.78±0.2a	7.75±0.3a	6.95±0.5efg	6.89±0.1gh	7.05±0.5cde	6.94±0.4fg	7.15±0.5c	7.09±0.1cd	7.47±0.2b	5.85±0.1k	5.75±0.2k	7.75±0.1a	7.71±0.2a	6.9±0.1gh	6.79±0.1h	6.93±0.1fg	6.92±0.3fg	7.09±0.1cd	7.01±0.5def
EC	1.21±0.02g	5.87±0.03b	5.99±0.1b	1.15±0.05g	1.18±0.06g	2.17±0.09g	2.78±0.07d	2.67±0.07d	3.35±0.06c	1.69±0.1f	1.74±0.09f	1.23±0.03g	6.51±0.02a	6.64±0.1a	1.35±0.15g	1.4±0.06g	2.24±0.04g	2.85±0.04d	2.89±0.11d	3.54±0.13c	1.89±0.1f	1.79±0.04f
Moisture	7.55±0.45gh	4.8±0.4k	3.06±0.41l	9.55±0.45de	11.2±0.4bc	9.05±0.3ef	7.63±0.33gh	11.16±0.3bc	7.04±0.4hij	14.33±0.3a	13.51±0.3a	7.36±0.3ghi	2.67±0.35l	1.07±0.03m	8.28±0.3fg	9.57±0.25de	7.54±0.3gh	6.57±0.29ij	10.27±0.0.3cd	6.29±0.0.35j	12.09±0.0.3b	11.3±0.4b
WHC	620±10bcdefg	565±15efg	550±20g	664±19abc	705±10a	610±10bcdefg	650±10abcd	627.5±7.5bcdef	657.5±12.5abcd	685±15ab	672.5±12.5ab	615±7.5bcdefg	395±7.5h	365±12.5h	596.5±21cdefg	615±21.5bcdefg	552.5±10fg	586.5±8.5defg	557.5±9fg	546.5±16g	645±11abcd	640±17abcde

Data in table represents the mean values of three replicates (n = 3) ± standard error and different letters represent significant differences declared by the LSD test at $p \leq 0.05$. Cu-Copper 300ppm; Ni-Nickel 100ppm; *T. asp-Trichoderma asperellum*; BC-rice husk biochar; EC-Electrical Conductivity; WHC-Water holding capacity.
